# Nurses’ views of patient- and family-centered care and its practices in peri-operative contexts in hospitals in Northern Ghana

**DOI:** 10.1186/s12912-024-01747-w

**Published:** 2024-02-06

**Authors:** Bernard Atinyagrika Adugbire, Portia Janine Jordan, Young Cornelle

**Affiliations:** Department of Nursing, Faculty of Medicine and Health Science, Stellenbosch University, Cape Town, South Africa

**Keywords:** Patient- and family-centered care (PFCC), Peri-operative contexts, Hospitals in Ghana, Nurses in Ghana, Benefits of PFCC

## Abstract

**Background:**

The purpose of the study was to explore the views of nurses on the concept of patient- and family-centered care (PFCC) and its practices in a peri-operative context in Northern Ghanaian hospitals.

**Methods:**

The study, using a qualitative explorative, descriptive, and contextual research design was conducted at six selected hospitals located in the Northern part of Ghana. Purposive sampling was used to recruit participants for individual and focus group interviews. Data were generated between March and May 2022. Data saturation was reached with 27 participants comprising 15 individuals and 12 members from two focus group interviews. All interviews were digitally recorded and transcribed verbatim and the data analyzed using thematic analysis.

**Results:**

Two themes emerged from the study, namely nurses’ understanding of the concept of PFCC and how they see the benefits of PFCC in a peri-operative context. Subthemes of the nurses’ understanding of PFCC included their perceptions of PFCC, the need for collaboration, supporting the patient’s family for better post-operative and effective communication, and PFCC practices in the peri-operative context. Subthemes for benefits of PFCC in the peri-operative context included nurse-related benefits, patient- and family-related benefits, and healthcare system-related benefits.

**Conclusions:**

The study revealed that the concept of PFCC is unfamiliar to the Ghanaian peri-operative context despite the positive perceptions exhibited by the nurses in terms of their understanding of PFCC and its related benefits to nurses, patients, and families as well as the healthcare system.

**Supplementary Information:**

The online version contains supplementary material available at 10.1186/s12912-024-01747-w.

## Background

To practice patient and family-centered care, the Institute of Medicine (IOM) which is a health arm of the National Academic of Sciences based in Washington, USA, identified six healthcare dimensions aimed at improving the healthcare system globally. These dimensions include (1) Patient-Centered Care (2) Provision of care that should consider the patient’s values, preferences, and desired needs (3) that care should be coordinated and integrated (4) there should be provision of information, communication, and education (5) that there should be provision of physical comfort (6) and that the care should be able to provide emotional support, devoid of fear and anxiety and must include the family [1, 2]. This call by the Institute of Medicine (IOM) to improve the healthcare system by way of attention to these six dimensions has led to a global increase in the practice of patient and family-centered care (PFCC) in hospitals and other healthcare settings.

However, the application of PFCC in peri-operative contexts has been perceived as both positive and negative [3, 4]. For instance, studies have pointed out that PFCC increases infection prevalence, since patients and families may not be able to observe infection control mechanisms during the care process [5, 6]. Furthermore, nurses also observed that PFCC in the peri-operative context may negatively affect hospital policies or protocols, such as visiting time and ward rounds, as these activities impede nurses’ work [5, 6]. Because of such negative perceptions about PFCC in the peri-operative context, nurses exhibit poor attitudes toward the implementation of PFCC and are often unwilling to engage with PFCC or accept PFCC implementation policies [7, 8]. This negativity towards PFCC occurs despite the existence of standardized models, guidelines, policies, and procedures to provide nurses with the necessary knowledge and skills to implement PFCC [9].

A study within a USA context explored the impact of PFCC on nurse-family interaction in the neurology unit and revealed that nurses perceived PFCC as challenging [10]. This could be because PFCC cannot be dealt with singly but must involve multiple stakeholders such as nurses, patients, family, and hospital managers interacting in a complex ward environment [10, 11]. The need thus exists for a well-designed unit within the hospital to coordinate the activities of PFCC, so as not to affect other care processes unrelated to PFCC practices. Some studies have shown that the best tool for the necessary coordination to implement PFCC would be to establish a unit purposely for PFCC practices within the peri-operative context [12, 13].

Despite the negative perceptions exhibited concerning PFCC implementation, it was found that the involvement of patients and families in the peri-operative context improves PFCC translation into practice [14]. Another study confirmed that patients and their families’ involvement in care results in the improvement of care, as family members assist in calming agitated or disoriented patients, ensure patients’ sense of safety and comfort, and also contribute to decision-making on behalf of unconscious relatives [15]. Other positive consequences include the ability to recognize the cultural needs of patients and families, the importance of the extended family’s support, the value of obtaining a more complete set of medical information about the patient from the family, and seeing patients as unique individuals with specific needs and preferences as part of a needed family support system [1].

In Southern Ghana, a study has indicated that nurses are familiar with the concept of PFCC but that PFCC principles, components, and dimensions are unfamiliar to the Ghanaian context, a situation that complicates the full implementation of PFCC [3]. The same study thus recommended the development of a context-specific PFCC model that will reflect the values and cultural norms of Ghana. Such recommendations were supported by a study that emphasized the importance of considering patients’ and family values, beliefs, and cultural and religious perspectives, for incorporation into the planning and the delivery of care to patients [16].

The provision of PFCC involves a multidisciplinary team such as nurses, patients, and families as well as clear aims and objectives to focus on the provision of a quality care experience by the patients and their families [17]. The advantages of PFCC in peri-operative contexts have been considered in three areas, namely nurse-related advantages, patient- and family-related advantages, and healthcare system-related advantages.

An integrative review of patients’ perspectives on PFCC revealed that in peri-operative contexts, PFCC is seen as a key indicator of successful surgery [18] while it has been argued that in peri-operative contexts, PFCC allows nurses to treat patients and their families as unique individuals with relevant values and norms. Thus, PFCC practices help nurses to identify patient individuality as well as the unique needs and preferences of patients. Such information can then be used to develop care plans to deliver patient-centered care which is beneficial to the recovery process [18–23].

In peri-operative contexts, the involvement of the patient and family has the potential to influence the outcome of patients’ conditions and improve the quality of care, which brings job satisfaction to nurses [24]. Subsequently, patients’ family members are important in becoming actively involved and collaborating in fundamental or basic care activities [14, 24] which combine physical, psycho-social, and relational dimensions of care normally provided by nurses [24, 25].

Basic care activities include attending to specific needs such as doing a simple dressing, mobility needs (such as early mobilization, and head-of-bed elevation), nutritional needs (such as encouraging oral intake or assistance in feeding), personal hygiene needs (such as mouth care, elimination, shaving, and styling hair and bathing), and social needs (such as companionship during the hospital stay) [26, 27]. Family involvement may thus significantly reduce the workload of nurses. Other benefits resulting from families’ involvement are the provision of basic safety prevention measures as a result of their physical proximity, and thus prevention of patient falls. Furthermore, families are present during surgical rounds to provide vital information to inform better treatment choices by the multidisciplinary team, and faster recovery of the patient [ 14, 29]. Nurses also get the opportunity to coach and train family members to acquire knowledge and new skills in performing these basic activities, as well as supervise family members’ activities [14, 28].

Since PFCC focuses both on processes of care and on relational aspects of care, it produces advantages for both patients and families [29]. The presence of the peri-operative nurse practicing PFCC provides a calming influence and prevents feelings of loneliness in the impersonal theatre situation. Patients appreciate orientation to the theatre environment and personal touch during surgery by the peri-operative nurse, attention which decreases pre-operative anxiety and leads to better surgical outcomes such as early recovery, effective postoperative pain control, and increased early mobility [30, 31]. Apart from increased patient and family satisfaction, implementation of PFCC brings significant benefits such as reduced mortality, improved patient care, quality care across settings, and reduction of cost incurred by patients and families [32].

Within the healthcare system, PFCC enables management to establish meaningful information exchange channels with patients and families. Because PFCC is a collaborative process, implementation thereof should lead to an informal analysis of all the expectations of management, patients, and families as stakeholders towards identifying and creating available services for patients and families [11, 33]. This leads to the consideration of a balance between patients’ and hospital needs on the one hand, and stakeholder preferences on the other [34].

Patient and family-centered care enables management to identify policies and regulate systems that are not in alignment with PFCC principles [11]. Thus, PFCC in the peri-operative context will bring flexibility in care provision and management of risks instead of rigidly following rules and regulations that do not favor PFCC.

Patient and family-centered care assists management in setting appropriate expectations to help the healthcare system move from task-focused care to evidence-based PFCC practices [11]. There must be a consistent capacity and encouraged creativity to provide PFCC and so provide quality of care in the health care system [11, 33]. To achieve this, the development of a PFCC framework and interventions aimed at addressing the issues related to PFCC at both managerial and staff levels seems paramount [35]. Drawing on relevant literature and emerging arguments the study explored the views of nurses on the concept of Patient and family-centered care and its practices in Northern Ghana. Though PFCC involves a multiplicity of stakeholders, nurses interact more with patients and their families in a peri-operative context as compared to the other stakeholders in the delivery of peri-operative care. Thus, the study focuses on nurses to explore their views of the practice of PFCC since they have more care experiences with patients and families than the rest of the stakeholders.

## Methods

### Design

A qualitative explorative, descriptive, and contextual research design [36, 37] was used to explore nurses’ understanding of the concept of PFCC and its practices in the peri-operative context in hospitals in Northern Ghana.

### Setting

Ghana is a West African country, which shares borders with Côte d’Ivoire to the west, Togo to the east, Burkina Faso to the north, and the Gulf of Guinea to the south. Ghana covers a total area of 238,533 square kilometers with a population of 29,463,643. There are sixteen administrative regions in Ghana, which can be zoned into three distinct regions, named the southern, middle, and northern zones. The Northern zone is further divided into five administrative regions. These regions include the Northern, North-east, North-west, Upper East, and Upper West regions. The study was conducted in the upper East region of Ghana. However, the Upper East region is the second largest region in Northern Ghana. This Upper East region shares borders with other neighboring countries, with the closest country being Burkina Faso. Consequently, the selected hospitals in the region also provide peri-operative care to people from the neighboring countries who are either staying in the region or are being referred from the health centers. As a result, the region has varied people who come from different cultural backgrounds. The inhabitants in the area are thus from different ethnic groups, with different cultural and religious perspectives about health and illness and obtaining health care. The Upper East region was selected for the study because of its cultural variability and ideas related to hospitalization and PFCC peri-operative care by the region’s different populations.

The study was conducted at two (2) municipal and four (4) district hospitals in the Upper East Region of Ghana. The hospitals are the municipal hospital in Bolgatanga, the War Memorial Hospital in Navrongo, the Builsa District Hospital in Sandema, the Bongo District Hospital in Bongo, the Presbyterian Hospital in Bawku municipality, and the Bawku West District Hospital in Zebilla. These are the major hospitals in the Upper East Region of Ghana that admit patients for peri-operative care. Several types of surgeries are performed in these hospitals and they are the largest in the region, all having the required staff to provide the needed peri-operative care to patients.

### Population and sampling technique

The target population for this study was nurses who were working in the peri-operative context at the six selected hospitals. These included surgical ward nurses, theatre nurses, and nurse anesthetists. These categories of nurses were purposively targeted at the selected hospitals due to their frequent interaction and mutual relationships with the patients and their family members during peri-operative care.

### Data collection instrument and procedure

Data was generated by two interview processes, namely individual and focus group interviews using a self-developed semi-structured interview guide with the same interview questions for each process as provided as a supplementary document. Both interviews were conducted in English. The interview guide was developed based on the objective of exploring the nurses’ perceptions of PFCC and determining their views on the benefits of practicing PFCC in the peri-operative context. The essence of using both individual and focus group interviews was to enhance data richness and ensure there is more in-depth information into their understanding of the concept of PFCC in the peri-operative context.

Individual interviews were conducted in hospitals with a smaller number of participants and were conducted for 30 to 45 min at places convenient to the individual participants. Each participant signed a consent form and their participation was voluntary as they could withdraw from the interview or decline the interview without any consequences. Pseudonyms were also used in reported data, such as participant (P1) for confidentiality purposes. These interviews were conducted between March and May 2022 at the four smaller selected hospitals. Data saturation was reached when participants provided similar responses and no new information surfaced. A total of 15 participants were finally interviewed. The fifteen participants who were interviewed individually included surgical ward nurses, nurse anesthetists, theatre nurses, and peri-operative nurses from the following facilities: Zebilla (4), Sandema (4), Navrongo (4), and Bongo (3).

Focus group interviews were conducted at each of the two different hospitals with a larger number of participants in their surgical units. Focus groups consisted of six (6) participants at each of the two larger hospitals. The first focus group interview took place at Bawku Presbyterian Hospital, while the second was conducted at the Bolgatanga Hospital. Each focus group involved six members comprising surgical ward nurses, theatre nurses, nurse anaesthetists, and peri-operative nurses. Thus, a total number of 12 participants.

The purpose of conducting the focus group discussion was to allow the participants from the two larger hospitals to discuss in detail the concept of PFCC. This information was to support the findings of the individual interviews and enhance the robustness of the findings. Both individual and focus group interviews were digitally recorded with the permission of the participants.

### Data analysis

Data generated from individual and focus group interviews were transcribed verbatim from audio to text. Data collection and transcription were done concurrently, in order not to lose relevant information and an inductive thematic analysis was applied to the content of the transcribed data [38].

An inductive thematic analysis approach was used to analyze the data. With the inductive approach, the authors read the data to make meaning of the data and allow the codes to emerge without imposing existing theoretical concepts.

Using the inductive thematic analysis, the lead author read the data for familiarization with the research data, generated initial codes, searched for themes in the data, reviewed, defined, and named the themes, and produced the result.

With familiarization, the lead author transcribed the data from both the individual and focus group interviews and then read the transcribed data several times to become familiar with the data. During this stage, the author reflected on initial ideas about the data without necessarily coding the data and took mental note of keywords.

The initial codes were generated by formulating the initial topics of the transcribed data using manual techniques. Data with similar codes were put into categories. After searching for initial codes, a search for themes was done by sorting the different codes into potential themes. These potential themes were then put into main themes and sub-themes. The themes were reviewed at the coding level by re-reading the transcribed data that fit each theme to ensure the data was organized in a coherent pattern. The themes were also reviewed to ensure there was a relationship between the themes. This ensured that the formed themes reflected the meaning of the whole data, and the preliminary findings were presented to nurses at the regional hospital, Bolgatanga, for their feedback. After this, the themes were defined and named to identify the importance of each theme and the portion of the data each theme defines. All discrepancies were discussed with the co-authors to agree on the final themes and sub-themes. These agreed-upon themes were then reviewed severally to ensure they fall within the objectives of the study. Thus, the merging of the data was done during data analyses, leading to the development of the themes and sub-themes. The authors discussed the themes and subthemes to ensure that the emergent themes truly reflected nurses’ views of PFCC in the peri-operative context.

### Rigor

The principles of trustworthiness namely credibility, transferability, dependability, and confirmability of the results were applied to enhance rigor in this study [39]. Credibility was enhanced through prolonged interaction with participants. Thus the lead author with his experience in a peri-operative context, spent more days and time interacting with nurses at the selected hospitals during data collection. There were follow-up questions for more clarification on salient issues raised. The transcripts were read several times to make meaning and enhance the coding of the data. The codes and themes were labeled and re-labeled to ensure that they represent the true meaning of the data. To ensure member checking, the lead author validated the audio recordings and transcripts by visiting the participants at the selected study areas for them to confirm the audio recordings with the transcripts. This was done during the second round of the interview to ensure that the data collected was a true reflection of the participant’s comments. The participants were purposefully recruited. Transferability was enhanced through a detailed description of the setting of the study and the whole plan for the study. To promote the dependability of the generated data, an external audit was done by the co-authors, while the same interview guide was used to generate data from both individual and focus group interviews. For confirmability enhancement, a field diary and records of the research processes have been kept and are available as an audit trail.

## Results

The participants’ demographic characteristics indicate the participants’ hospital name, the letter (P, 2, etc.) representing each participant, gender, age, level of education, participant area of specialization, and spoken language. The participants’ demographic characteristics have been illustrated in Table 1 below.

**Table 1:** Demographic characteristics of participants

Hospital Name	Participant (P)	Gender-Male-M Female-F	Age	Level of education	participants’ Specialization	Spoken Language
**Zebilla**	P1	M	23	Undergraduate	Surgical ward	English
	P2	M	25	Undergraduate	Peri-operative	English
	P3	F	24	Nursing Training college	Surgical ward	English
	P4	M	30	Undergraduate	Theatre Nurse	English
**Sandema**	P1	M	32	Undergraduate	Theatre Nurse	English
	P2	F	35	Masters	Surgical ward	English
	P3	F	34	Undergraduate	Peri-operative	English
	P4	M	24	Diploma	Surgical ward	English
**Navrongo**	P1	F	30	Undergraduate	Theatre nurse	English
	P2	M	45	Undergraduate	Nurse Anaest.	English
	P3	F	40	Masters	Peri-operative	English
	P4	F	25	Diploma	Surgical ward	English
**Bongo**	P1	M	34	Undergraduate	Peri-operative	English
	P2	F	42	Masters	Theatre Nurse	English
	P3	M	26	Undergraduate	Surgical ward	English
**Bawku Presbyterian**	P1	F	32	Undergraduate	Theatre nurse	English
	P2	F	33	Undergraduate	Nurse Anaest.	English
	P3	M	27	Undergraduate	Peri-operative	English
	P4	M	35	Masters	Surgical ward	English
	P5	M	38	Masters	Surgical ward	English
	P6	M	26	Undergraduate	Peri-operative	English
**Regional hospital,Bolgatanga**	P1	M	28	Undergraduate	Theatre nurse	English
	P2	M	36	Masters	Nurse Anaest.	English
	P3	F	39	Masters	Peri-operative	English
	P4	M	28	Undergraduate	Peri-operative	English
	P5	M	25	Diploma	Surgical ward	English
	P6	M	28	Undergraduate	Theatre Nurse	English

Of the total of 27 participants who participated in the study, eight (8) had specialist qualifications in peri-operative nursing (PN), three (3) were nurse anesthetists (NA), seven (7) were theatre nurses (TN) and nine (9) were surgical ward nurses (SWN). These surgical ward nurses have been practicing for the past three years and above and have the rank of nursing officer to senior nursing officer in the nursing profession with an age range being thirty years and above. They therefore have rich experiences in peri-operative care. The views of the individual nurses and the focus group discussion were similar as presented as follows.

### Nurses’ understanding of the concept of patient- and family-centered care

The participants had different understandings of what the concept of PFCC meant to them within the peri-operative content. Their understanding of the phenomenon included their views about what the practice entails and its value, the need for collaboration, information sharing and communication, and PFCC practices in the peri-operative context as the subthemes of this first main theme.

### Nurses’ understanding of PFCC in the peri-operative context

The study results indicated that participants had a variety of understandings of what the concept of PFCC entailed, and the potential value of PFCC in the perioperative context. Some participants were of the view that PFCC would mean that the patient and their family members would take responsibility for their health and not depend on the nurses only. PFCC was viewed by one participant as rendering respectful and dignified care to patients and their families, while another participant understood that PFCC would recognize the unique cultural needs, values, and roles of patients and families and the need to support them better during care delivery. The verbatim quotes below illustrate participants’ views as revealed by this sub-theme.

*“I understand PFCC practice to be a laudable idea because PFCC is where the patient and family will take center stage in their care or activities toward their care. So, I think it is a nice program that will focus on the patients and families taking responsibility for their health and not only the nurses.”* (TN2).

“*I perceive PFCC to be a good practice because PFCC is where the care nurses would render to patients and their families would be respectful and dignifying taking into consideration the patient and the family opinions. I think it will be beneficial to the patients and their families”* (SWN8).

*“I think PFCC practice in the peri-operative context is the care that will help nurses to recognize the unique cultural needs, values, and roles of the patients and families during care, such as calming down the patients and providing the needed support”. (*PN3)

However, some participants expressed contradictory views on the value of the concept of PFCC in the peri-operative context. They indicated that such practices would negatively impact already poor infection control mechanisms, leading to increased infection rates in patients and families. To illustrate:

One participant stated: *“With this, our system of poor infection control mechanisms, practicing PFCC in the peri-operative context will bring more infections to the patients and even to the relatives” (SWN6).*

Another participant added: *“Our infection control system is not good at all, so I am of the view that practicing PFCC in the peri-operative context will rather bring more infection to patients and their relatives”(TN1).*

### Collaboration between nurses, patients, and families

Participant nurses viewed PFCC as a collaboration between the management, nurses, patients, and families to promote relationships and to implement PFCC in the peri-operative context. The following verbatim quotes illustrate the views of these participants.

“PFCC involves collaboration between the management, nurses, patients, and their families during nursing care for mutual benefits of all parties involved” (SWN1).

“PFCC is seen as a cordial relationship between patients, nurses, and the surgical team to enhance effective peri-operative care” (SWN2).

“\I understand PFCC to be a good collaboration between the nurses, patients, and their relatives as well as their community members regarding PFCC. If there is collaboration among all these people the implementation process will be smooth”\ (PN3).

### Effective communication between nurses, patients, and families

The participants understood that PFCC provides for effective communication between nurses, patients, and families, which would improve patient care delivery. Some illustrative comments:

*“PFCC practice involves diligent and timely communicating so that all the parties will be able to prepare adequately before whatever procedure to be carried out so that it will also protect all parties legally, and result in better peri-operative care”*. (TN1)

*“PFCC is just all about making sure that whatever you are going to carry out for the patient, the patients and his or her relatives are well aware, necessary information is communicated to them, and then you also allow them to ask questions, you clear their doubts and then you give them the maximum cooperation”.* (PN5)

Furthermore, some participants considered PFCC as helping to consider the patients’ and families’ personal preferences, using their preferred language and avoiding medical jargon, thus ensuring maximal inclusion of patients and their families. To illustrate:

“*Peri-operative nurses must listen to the patient throughout this phase where they have contact with the patient. We are also obliged to answer questions about the patient’s conditions to the patient and their families and respond to personal preferences using the appropriate language to make them feel that they could influence their care”.* (SWN6)

*“Sometimes using a simple language devoid of medical jargon and allowing a patient to respond are effective ways of communicating to the patients and families to understand and participate in care during PFCC practices”* (PN5).

### Supporting the patient’s family for better post-operative care

The participants also indicated that PFCC means that information should be shared between nurses, patients, and families for improved peri-operative care. For families that need better information, this would support them to care for their relatives after discharge. The following verbatim quotes highlight such views.

*“The family too will be shared with necessary information on how they should handle the wound, and how to assist the person to also take the medication that the person is supposed to take and to ensure that whatever the patient is asked to do on discharge is done because if the family is not given that role to play, patients can go to the house and will not follow the instructions”* (SWN4).

*“I think PFCC practice will enable the surgical team to share information in detail, what the entire surgery is all about, the risks and benefits of the surgery to the patients and families so that informed consent can be secured for a successful operation.* (PN7).

### Patient- and family-centered practices in the peri-operative context

Participants had varied views on the practices of PFCC in the peri-operative context. Some participants indicated that, at their hospital, the full continuum of PFCC is not being practiced as nurses only provide limited information to the patients and their families. As illustration:

“*I can confidently say that PFCC practice in this hospital is absent in all the units—orthopeadic, general ward, and theatre. We only provide the requisite information to the patients and their families when we need something”* (SWN 6).

*“Some of the nurses do not even know that there is something* (PFCC) *like that. Some of the nurses are ignorant about it and never even thought of practicing PFCC during their care processes”* (TN 2).

Because some surgical patients and their families do not get the necessary information about the surgeries, it seems to make them more confused and anxious, according to the participants. To illustrate

*“In my considered view PFCC is not being implemented well as a lot of the clients come into the ward for surgery without knowing all the surgical team members and also do not know exactly what type of surgery will be performed on them as a result, they look confused and anxious”.* (SWN2).

*“For this hospital, I will say we are to some extent focusing on only the patient but the family to some extent is always left out. Because we have instances where a patient is coming to the theatre and we lock the patient’s relative outside and push the patient inside. I think we are not being that responsive in that regard”* (NA2).

### Benefits of patient-and family-centered care in the peri-operative context

Participants thought that practicing PFCC would bring benefits, not only to the nurses, but to the patients and families and the hospital, and the health system in general. Thus, the benefits of PFCC in the peri-operative context emerged as the next main theme, with subthemes that included nurses-related benefits, patient and family-related benefits, and healthcare system-related benefits.

### Nurses-related benefits

Some participants opinionated that PFCC will help them to tailor care to patient needs, and provide respect and dignity, with the benefits of improving patient recovery and ultimate reduction of nurses’ workload. To illustrate:

*“Practicing PFCC in peri-operative nursing will allow the nurses to focus more on patients and their families, hence, the care rendered is tailored to their needs and that will improve the patient recovery and reduce our workload*”. (SWN8).

*“When PFCC is practiced in the peri-operative context, it will help nurses to treat patients and families with respect and dignity and according to their needs and expectations and this will bring satisfaction to us (nurses) since they will be participating in the care*”. (PN7).

*“When PFCC is practiced, some basic fundamental care such as feeding, bathing, bowel, and bladder care, etc. will be performed by the patient’s families, thus reducing our workload”* (SWN 2).

Some participants mentioned that practicing PFCC in a peri-operative context will minimize surgical care errors and infections, thereby reducing their workload of doing daily dressings to infected wounds in the ward.

“*The practice of PFCC will help us minimize surgical errors and thus reduce postoperative wound infection which will help the nurses to avoid daily dressing of infected wounds after the operation, thereby reducing their workload”.* (SWN6).

### Patient and family-related benefits

Participants indicated that implementing PFCC in the peri-operative context will benefit the patients, including reducing their anxiety and fear. As illustration:

*“With the nature of operations, there is anxiety, fear, so with PFCC practices, patient and family needs would be understood and supported with education and counseling to ease their fears and anxiety”.* (PN6)

Furthermore, participants indicated PFCC would benefit the patients and families as they would be orientated to the theatre environment, which will reduce their anxiety and fear thus calming the patient down before the operation. Two comments as illustrated:

*“When we practice PFCC, we will orient the patients and families to the theatre environment, so when a patient goes inside the theater and sees strange things, he would not be worried or afraid because he knows that those things inside the theater are just there to help him or her go through the operation. So, the patient would be relieved or would not have any anxiety”.* (TN3)

*“I think that practicing PFCC in a peri-operative context where the patients and their families will be present in theatre during operation, will help reduce their anxiety and fear and will enable them to ask questions that will further clarify their mind, so it will help them”* (TN2).

Other participants stressed that PFCC has the potential to increase patients’ and families’ satisfaction, reduce mortality, improve patient care quality across settings, as well as reduce cost. Two illustrative comments:

*“I think that practicing PFCC would actually help to reduce mortality post-operatively, increase patients and family’s satisfaction of care and improve the overall patient outcome and will even reduce surgery cost” (*TN5).

“*I feel when PFCC is implemented, well, it will lead to a better patient outcome that will result into more discharges, better home care by relatives, and an overall reduction in care cost”*. (SWN5)

### Health system-related benefits

Some participants indicated that the practice of PFCC in a peri-operative context would help the management of the various hospitals and the Ministry of Health (MOH) to develop policies to address the concerns of patients and families based on issues that may arise during PFCC practices. To quote some illustrative comments:

*“I think PFCC will help with certain ideas that the Ministry of Health (MOH) and the government want to be implemented since there is a public concern about poor nursing care at the hospitals in the country. So, I think if PFCC is implemented it will benefit the health system in areas such as development policies to effectively address the patients’ and families’ concerns”.* (SWN8).

*“I think if PFCC is practiced in our setting…this will also let the team explain everything to the family: about what the team is going to do on their patient and assure them of everything that will be taken care of, and in fact, it will improve the quality of our health care system”.* (NA2)

Furthermore, some participants indicated that PFCC will benefit the healthcare system since it will help identify aspects that need to be addressed in education efforts. To quote one theatre nurse:

*“Practicing PFCC will benefit the health care system such that it will help in diagnosing issues in the family that the patient has not mentioned but during your interaction, they can bring them out. So, PFCC will help the team to encourage the family and educate them on those issues that are affecting the care process, thus improving the overall health care system” (TN6).*

## Discussion

Participants in the study expressed different views regarding their understanding of the overall concept of PFCC and its potential value in the peri-operative context. Overall, PFCC was viewed as a collaboration between the management, nurses, patients, and their families to promote good interpersonal relationships and implement PFCC in the peri-operative context. Furthermore, implementing PFCC means that valuable information could be shared between nurses, patients, and families for improved peri-operative care. The study’s findings emphasized that respectful and dignified care could be rendered to patients and their families by implementing PFCC and that patients and family members may be in a better position to take more responsibility and provide support toward patients’ health and well-being.

These conceptual understandings of PFCC largely correspond with a study that reported on PFCC, comprising four core concepts [39]. These concepts include nurses demonstrating respect and appreciating the dignity of patients and their families; ensuring information sharing between nurses, patients, and families; enhancing patients’ and their families’ participation in care; and promoting effective collaboration between nurses, patients, and their families during care.

Furthermore, this study’s findings provide a unique contribution to the study of PFCC that has not been adequately captured by existing literature. For instance, nurses stress the importance of identifying unique cultural needs, values, and roles of patients and families that could be considered, which will allow nurses to support them better throughout the perioperative journey in African countries such as Ghana. These findings are affirmed by another Ghanaian study [16] which explored nurses’ perspectives on the needs of surgical patients during the peri-operative period. Findings from this 2020 study indicate that patient and family values, beliefs, and cultural and religious perspectives should be considered and incorporated into the planning and delivery of patient care.

Also, varied views regarding the practice of PFCC in the peri-operative context emerged. Whilst some participants indicated that PFCC is unfamiliar to the Ghanaian perioperative context, and thus, that PFCC is not being practiced at all, others felt that PFCC is being practiced but not to its fullest potential as in Western countries. This variation was also illustrated by a 2019 study in Southern Ghana [3] to assess parents’ perceptions of family-centered care for children hospitalized through road traffic accidents, indicating that PFCC principles, components, and dimensions may be less familiar within the Ghanaian context. It may thus be more challenging for nurses to practice PFCC to its fullest potential in the peri-operative context in Ghana.

What also became clear was several controversial ideas about the value of PFCC. Concerns that PFCC in the peri-operative context would negatively impact already poor infection control mechanisms, leading to even more infections in patients and families were noted. Other studies [5, 6] support this view by stating that patients and families may not be able to observe infection prevention mechanisms during the care process. This is not a unique stance, as more studies [7, 8] indicate that nurses were also of the view that implementing PFCC in the peri-operative context will affect hospital policies or protocols and might increase infection rates. Such negative views of PFCC in the peri-operative context cause nurses to exhibit poor attitudes toward PFCC implementation, with an unwillingness to engage with PFCC or accept PFCC implementation policies [7, 8]. It is therefore recommended that nurses need to engage with the patients and families by educating them on infection control mechanisms that could help reduce the rate of infection.

Those participants who valued the practice of PFCC in the peri-operative context thought that practicing PFCC would contribute to the welfare of patients and their families since they would be actively involved in their care. This corresponds with findings from previous studies which indicated that PFCC helps to calm agitated and disoriented patients during admission to promote active participation in such care [1, 14, 15].

What was further revealed by the present study is that PFCC requires collaboration to achieve optimal patient care, similar to previous studies which indicated that collaboration between nurses, patients, and their families in policy and program development, implementation, and evaluation as well as in the professional development of nurses are key components to the successful implementation of PFCC [40, 41].

The successful implementation of PFCC in the peri-operative context, as highlighted by the present study, seems to require effective communication between nurses, patients, and families to facilitate the documentation of relevant information that can be used to improve the care process, though there may be other factors such as language barrier, health beliefs and cultural values that could impede the implementation process. Similar findings [41–43] emphasized that effective communication with patients and their families enhances the documentation of relevant information obtained for them to effectively plan for ideal care outcomes for both patients and families. This implies that steps should be taken to overcome the language barrier, health beliefs, and cultural values of the patients and families to successfully implement PFCC.

In conjunction with effective communication strategies are nurses’ keen interest in listening to patients and families throughout the care period. Nurses should answer questions asked by the patients and families and respond to the personal preferences of the patients and families. This is in congruence with earlier findings [44–46] whereby the importance of verbal and non-verbal cues was emphasized for nurses to understand patients and families and assist in overcoming physical, psychological, and social barriers affecting their participation.

In addition, effective information sharing between nurses, patients, and families was emphasized as fundamental to PFCC practices in the peri-operative context in the present study, as it empowers patients and families in the development of self-care strategies towards independence once discharged. These findings are in tandem with previous findings which revealed that nurses must partner with the patient and family to tailor strategies for self-management of care that are based on the patient and family characteristics and preferences [43, 47].

Patient and Family Centered Care inevitably introduces certain benefits to nurses, patients, families, and the overall health system as identified in this study. These findings are corroborated by Clay & Parsh [17] who emphasized that PFCC’s clear objectives, with a focus on the quality of care, are beneficial to patients and families, nurses, and even the health system.

In terms of the benefits of PFCC to nurses, the study indicated that this practice will allow nurses to focus more on patients and their families by showing respect and dignity since they participate in the care. Patient and Family Centered Care thus allows nurses to render care that is tailored to their needs and thus improves patient recovery, leading to nurses’ satisfaction with the care rendered. Through following the processes of PFCC nurses can identify the individuality of the patient, their unique needs and preferences, and use this information for the development of a patient-centered care plan that is beneficial to the recovery process and resultant increased satisfaction to nurses [18–23]. Further benefits of PFCC include a decrease in surgical errors during surgery which would lead to a reduction of post-operative wound infection. This will potentially reduce daily wound dressings in the surgical ward. However, contradictory opinions exist, where the idea is that PFCC would result in poor infection control and increased wound infection as a result of patient and family participation in care [5, 6].

Other identified benefits of PFCC include effective education of patients and families during peri-operative care to reduce anxiety and fear amongst patients and families. Similarly, Masry’s study [30] indicates that PFCC improves the education levels of the patient and family and eventually leads to the reduction of fear and anxiety on their part. Furthermore, in the current study, PFCC was seen as a practice that will help reduce post-operative infections, increase patients’ and families’ satisfaction with care, improve the overall patient outcome, and even reduce surgery costs. This view of PFCC corresponds with its benefits as highlighted by a study [32].

Finally, the study identified that, overall, the Ghanaian healthcare system would benefit when PFCC is being practiced because it would create information for the Ministry of Health (MOH) to develop policies to effectively address the patients’ and families’ concerns, leading to an overall improvement of the healthcare system. Such benefits are also confirmed by the observation that PFCC enables management to identify beneficial policies and system regulations following the implementation of PFCC [34, 48].

Management at hospitals will thus benefit from PFCC, as it will help them to diagnose hidden issues in the healthcare system and within families that have not been recognized before but are affecting the provision of care to the patient. Similarly, some studies [11, 33] showed that PFCC implementation would lead to an informal analysis of management, patients’ and families’ expectations towards available or new services needed within the health care system. This implies that PFCC implementation would allow management to assess the cost of care and nurses’ needs to support PFCC by responding to patients’ and families’ preferences and needs, allowing creativity within the healthcare system, and thus improving the quality of patient care and patient safety.

### Limitations of the study

The smaller number of participants at the selected hospitals was identified as a limitation. Thus, this probably affected the specific practices of PFCC at the selected hospitals, hence, the study’s findings cannot be generalized. Another limitation was the exclusion of patients and families in the study. Their inclusion could have brought in different perspectives of PFCC. However, the study provided rich qualitative findings that could potentially benefit peri-operative health contexts.

## Conclusion

In conclusion, the study explored nurses’ views on the concept of PFCC and its practices in the peri-operative context in Northern Ghana. Specifically, the study explored nurses understanding of the concept of PFCC and the importance of practicing PFCC in the peri-operative context. Although the idea of PFCC was considered an unfamiliar phenomenon to participant nurses, they were able to indicate that PFCC is mainly about providing nursing care to patients through collaboration, communication, and information sharing between nurses, patients, and families. Nurses viewed PFCC as providing opportunities for them to render peri-operative care to patients and families in a dignifying and respectful manner. Patient and family-centered care thus enables nurses to provide health care that takes into consideration the preference of patients’ needs, cultural and religious values, and norms. Apart from these, PFCC practices would benefit nurses in areas such as reduction in workload, post-operative infection, and improvements in surgical outcomes. At the same time, patients and their families could benefit in terms of knowledge acquisition to carry out fundamental care, and familiarity with the surgical environment leading to a reduction in fear and anxiety. PFCC practices could also assist in the formulation of policies at the national and regional levels to promote PFCC in the peri-operative contexts.

Despite these benefits, some nurses indicated that practicing PFCC will further compromise the already existing poor infection control mechanisms which may further bring more infections to the patients. They therefore seem reluctant to practice PFCC in peri-operative contexts though they are aware of its potential benefits to the nurses, patients, families, and the Ghanaian health system. This has implications for the management of hospitals in Northern Ghana, as they need to formulate local policies on PFCC and train nurses, patients, and families to practice PFCC. Also, the Nursing and Midwifery Council of Ghana needs to review the various curricula used for the training of nurses by incorporating the concept of PFCC into the curricula to train nurses to improve on the practice of PFCC. The findings will inform the Ministry of Health and Ghana Health Service to implement nationwide policies that will empower the implementation of PFCC in the peri-operative context.

### Electronic supplementary material

Below is the link to the electronic supplementary material.

**Supplementary Material 1:** Semi-structured Interview guide

## Data Availability

The data and materials of the study would not be shared though the data are with the corresponding author. This is to ensure that the participants’ anonymity and confidentiality are maintained since the participants provided sensitive information. Thus, the data are safely kept to protect the integrity of the participants.
