# Supplementary material for: Nurses’ views of patient- and family-centered care and its practices in peri-operative contexts in hospitals in Northern Ghana
Source: BMC Nurs. 2024 Feb 6;23:97. doi: 10.1186/s12912-024-01747-w (PMC10845520; doi:10.1186/s12912-024-01747-w)
Supplement: Supplementary file 1 — Supplementary Material 1: Semi-structured Interview guide [file 12912_2024_1747_MOESM1_ESM.docx]

**Annexure A**

**Semi-structured Interview guide for nurses in the peri-operative context**

**RO2: Explore and describe the perceptions of nurses on PFCC in peri-operative context**

**RO3: Explore and describe the barriers and facilitators to implementing PFCC in peri-operative context.**

- Tell me what do you understand by the concept PFCC.
- How do you perceive PFCC in the peri-operative context?
- What is your knowledge regarding PFCC in the peri-operative context?
- Elaborate on any PFCC models, best practice guidelines or any other evidence that you would use to guide you on the implementation of PFCC in the peri-operative context.
- How do you feel about implementing PFCC in the peri-operative context?
- Do you have any recommendations for how and what content related to PFCC can be used for an educational intervention in the peri-operative context?
- What are the barriers to implementing Peri-operative PFCC in the peri-operative context?

Probe

Stakeholders (management, patients and family members, community barriers)

- What do you think are the facilitators to the implementation of PFCC in the peri-operative context?
